# Effects of Environmental Conditions and Composition on the Electrical Properties of Textile Fabrics

**DOI:** 10.3390/s19235145

**Published:** 2019-11-24

**Authors:** José Torreblanca González, Raúl García Ovejero, Álvaro Lozano Murciego, Gabriel Villarrubia González, Juan F. De Paz

**Affiliations:** Expert Systems and Applications Lab, Faculty of Science, University of Salamanca, 37008 Salamanca, Spain; torre@usal.es (J.T.G.); loza@usal.es (Á.L.M.); gvg@usal.es (G.V.G.)

**Keywords:** dielectric properties, resistivity, conductivity, tissue, smart-textiles, textile materials, relative humidity, temperature

## Abstract

In our day to day life, the environmental conditions, and especially the temperature and humidity of the air that surrounds us, go unnoticed. However, in many cases, these parameters play an important role in the use of materials since they modify their electrical properties. It is necessary to predict what this behaviour will be as these environmental conditions can introduce or improve desirable properties in the material, especially of textiles. The nature of these is to be dielectric, and therefore have a minimal DC electrical conductivity that is currently impossible to measure directly, so a methodology has been proposed to obtain the DC electrical resistivity through the method of discharging a condenser. For this purpose, a system was developed based on a static voltmeter, a climatic chamber and a control and data capture units. In order to validate the proposed system and methodology a study using both is described in this work. The study made it possible to verify that the most influential factor in establishing the values of the electrical parameters of a textile material is the nature of the fibres of which it is composed, although the influence of environmental conditions in fibres is also significant.

## 1. Introduction

Understanding the electrical properties of materials and identifying their characteristics, possible applications, and ability to mix them in with other materials at the nanoscale is a matter of great interest to several industries; especially in the textile industry [1]. In this area, all types of textiles are important, but the so-called “smart textiles” [2] are the subject of special interest and research. Smart textiles are defined as those capable of altering their nature and modifying some of their properties in response to the action of different external stimuli, physical or chemical, mainly with the aim of conferring additional benefits to their users [3]. The advantages that these textiles present compared to conventional textiles are possible thanks to the incorporation of various devices—fundamentally electronic [4]—into the textile and they work in combination with other technologies such as nanotechnology, microelectronics or biotechnology [5] to obtain these results. These technologies are not only aimed at the textile field but are also an active research focus for various fields, including commercial, medical, military and aerospace, because smart tissue sensors offer technological possibilities that are not possible with conventional electronics only [6]. These added values brought about by the incorporating of technology can make textiles very useful, fun, supporting, protecting or even lifesaving. It is, however, essential for the comfort, acceptance, and functionality to make the integration of electronics as unobtrusive as possible. One elegant and unobtrusive method of integration is to have the circuitry included in the textile and components mounted to this circuitry [7]. In order to realize this integration, all possible properties of the elements involved must be known, including the properties of the base fabric; and the electrical properties, which must first be analyzed. For this electrical analysis, the term “electrical conduction” is the most important and to evaluate it, the term "electrical conductivity” (*σ*) is used, which is defined as the relationship between the current density and the electrical field in a material (the unit in the international system is the Siemens per meter, S/m) [8]. This value is always positive and may or may not be uniform; that is, it depends on whether its value varies or not according to the field applied. If it does not vary, they are called "linear” or “isotropic” media or materials, and if they do vary, they are called "non-linear” or “anisotropic” media or materials [9], but if the electric field is constant, this electrical conductivity must be unique and the only way of evaluating it varies since it cannot be obtained through suitable measuring instruments. The electrical parameter that directly measures the measuring instruments is the electrical resistance of the material, a parameter that is directly proportional to the “electrical resistivity” (value that is inverse to that of the electrical conductivity). This last term is the one used because it is the property of the material that is considered constant under controlled environmental conditions and independent of the measurement technique used [10]. In the textile base part and due to the high resistance of most textile materials and the difficulty to measure it, the evaluation of this electrical parameter is of particular interest as the resistivity of textiles cannot be easily measured and a number of different methodologies have been developed in different areas over the last few years to obtain electrical parameters related to electrical conductivity [11,12,13]. Amongst the different methods used in the analysis of electrical resistivity (ρ, Ω cm) of dielectric materials, the method of parallel bars by capacitor discharge is utilized for this study.

It is worth clarifying that it’s not only due to the incorporation of smart textiles into the market that electrical parameters are studied, there are also other issues related to this subject, such as the generation and dissipation of static electricity [14]. More importantly, in recent years, due to the problems they are causing in the manufacture of textiles because of the incorporation of new synthetic fibres and the use of increasingly fast machines [15,16].

The objective of this study is to establish a methodology based on one of the existing procedures for obtaining the DC electrical conductivity of dielectric materials under specific environmental conditions. Once the methodology is established, conductivity values will be obtained under different environmental conditions to evaluate the influence of environmental conditions on this electrical characteristic. To this end, a measuring system is proposed in this work in order to implement this methodology.

The paper is structured as follows: Section 2 explains the background topics and the method employed in this work. Section 3 presents the proposed methodology for obtaining electrical conductivity of fibers under different climatic conditions, and the measurement system proposed for this purpose. Section 4 describes a case study for validating the proposed system and methodology. Section 5 displays and discusses the results of the case study. Finally, Section 6 outlines the main conclusions extracted from this work. 

## 2. Materials and Methods

Numerous authors have addressed the issue related to the measurement of electrical resistivity in different areas [17,18,19]. In the particular case of the textile world, several methodologies have been defined and used to obtain some electrical parameter, especially electrical resistivity [20,21,22]. There are different classifications depending on the range of values of this parameter; textiles have been classified into four large groups according to their utility [23]: insulators (do not allow electrical conduction, $\rho >\;10^{12\;}\Omega \;\mathrm{cm}$), antistatic (provide antistatic protection, $10^{7}\;\Omega \;\mathrm{cm}\;<\;\rho <\;10^{12\;}\Omega \;\mathrm{cm}$), electrothermal (heat generators, $10^{3}\;\Omega \;\mathrm{cm}\;<\;\rho <\;10^{7}\Omega \;\mathrm{cm}$) and conductors (data transmission, $\rho \;<\;10^{3\;}\Omega \;\mathrm{cm}$).

Within these classifications, the antistatic type is significant in the textile world which has resulted in the creation of several standards relied upon in this field [24,25,26]. These standards include antistatic textile materials, and for a textile to be classified with this property it must exceed specific values indicated in this standard. For the purpose of qualifying for these standards the tests are carried out under fixed environmental conditions, without taking into account the changes that occur when these conditions change, a circumstance that will be verified to substantially modify the electrical conductivity of the material. In these standards, despite the continuous technological advances that allow the direct measurement of the resistance of materials of higher value, it is still not possible to measure the entire range of resistances existing in materials. Due to this circumstance, materials with high electrical resistivity require special attention since an indirect measurement method is required.

Among the commonly accepted methods for obtaining electrical resistivity of dielectric materials, the fastest and most reliable method for obtaining values of this parameter is the static voltmeter method by capacitor discharge. This method is employed in the present work and already used in the literature [27,28]. Throughout history it has been found that there are several factors that modify the electrical resistivity, which can be divided into two large groups; internal and external factors. On the one hand, the internal factors related to the composition, both the type of fibres of which it is formed, its proportion and the structure (warp and weft). On the other hand, external factors, such as temperature, relative humidity of the air and the frequency (when referring to alternating current—AC). At present, two of these external factors are the subject of studies by researchers: the frequency for electromagnetic protection [29] that is out of the scope of this study and the environmental conditions for the behaviour of electrical conductivity [30] that are addressed in this work.

Among the current measurement methods, the kind of current used in these methods is differentiated: alternating current (AC) or direct current (DC). This work focuses on the study of methods that use DC since the electrical properties analyzed are those mainly used in the development of fabrics for Smart Textiles.

In the experimental procedure described in this paper, it is necessary to differentiate and determine two different aspects when obtaining the results of the electrical conductivity of the textile material: the measurement method and the equipment used.

**Figure 1:**
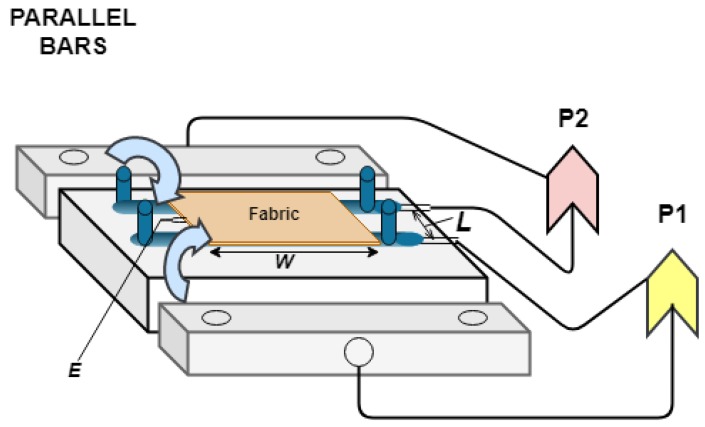
Diagram of the connection of the test tube holder.

The measurement method used is an indirect method for the reasons discussed above, that is, the electrical conductivity of the textile material calculated through a series of equations that are different according to the measurement technique used, but whose initial acquisition parameter always is the electrical resistance of the textile material. There are several techniques to obtain this first variable of the insulating materials, of which the measurement method used is the so-called “parallel bars” [31]. This consists of obtaining the DC electrical resistance of a tissue specimen between two parallel bars, which are integrated into a sample holder and are subject to a voltage difference, as shown in Figure 1.

**Figure 2:**
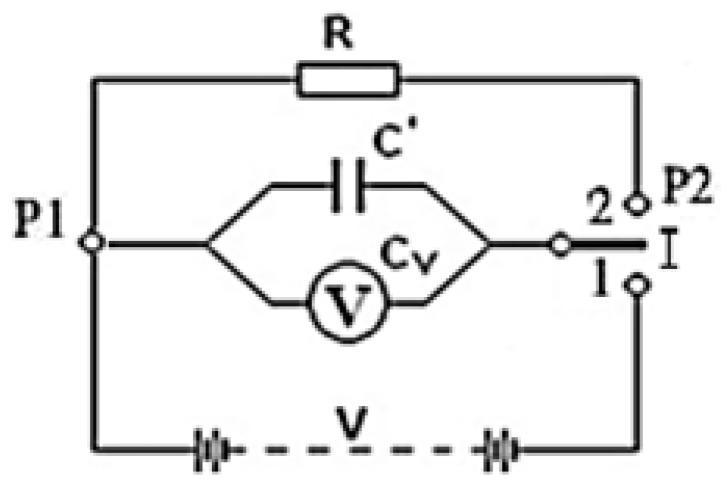
Equivalent circuit of the static voltmeter.

During the test, the characteristics that influence the values of the electrical resistance must be kept constant, for this reason, the probe holder was placed inside a climatic chamber that controls these variables automatically and instantaneously. The initial potential-difference between the bars (between terminals P1 and P2), called V0, is provided by a device called “static voltmeter” and its value is measured throughout the test without interference. Despite the internal complexity of the device, its electrical equivalent circuit is simple, as shown in Figure 2. 

**Figure 3:**
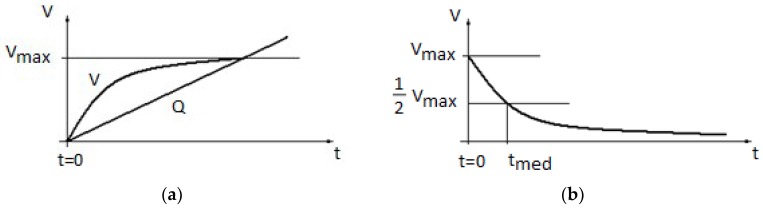
Charge process (**a**) and discharge (**b**) of the capacitor.

Through this electrical equivalent circuit, it is possible to obtain the value of the electrical resistance of the textile specimen (R) using the method of discharging the condenser. It consists of initially providing voltage V0 to capacitor C when placing “switch I” in position 1. It is essential to apply this measurement method from the same starting conditions, that is, initially the capacitor must be charged up to its maximum voltage (Vmax); knowing that the charging time depends on the capacity of the capacitor (C) and the electric charge (Q), as shown in Figure 3a. This translates into the initial waiting time until the capacitor is fully charged. Once this value is reached, the condenser is discharged through the textile material as shown in Figure 3b, and for this, the “switch I” is changed to position 2. This step involves a very high time until its total discharge. Therefore, the electrical resistance of the textile material will be obtained from the values of the half-discharge time ($t_{med}$) [32].

Analysing the electrical equivalent circuit of Figure 2 when “switch I” is in position 2, Equation (1) is derived to obtain the value of the DC volumetric electrical resistance:

(1) $$R=\frac{t}{C\;\mathrm{Ln}\left(V0/\mathrm{Vf}\right)\;}$$

where *R* is the DC electrical resistance in (Ω), *t* is the discharge time of the capacitor from the initial voltage to the value of the final voltage in (s), C is the capacitance of the equivalent capacitor provided by the manufacturer in (F), V0 is the initial voltage of the capacitor in (V) and Vf is the final voltage of the capacitor in (V). The parameter t is the one acquired in the test and the parameter *R* is the “specific” (volumetric) resistance of the textile obtained due to the tissue holder (it has been designed to achieve this type of DC electrical resistance).

When using the method of measuring “parallel bars” the value of the DC volumetric electrical resistivity of the textile is calculated by Equation (2) (which is a function of the resistance *R* and the dimensions of the specimen):

(2) $$\rho_{DC}\;=\frac{R\;A}{L\;}=\frac{R\;E\;W}{L\;}$$

where $\rho_{DC}$ is the DC volumetric electrical resistivity in (Ω cm), *R* is the DC electrical resistance, A is the surface of the textile section in (cm^2^), L is the length between the electrodes in (cm), E is the thickness of the textile sample in (cm) and W is the width of the textile sample in (cm). The values of the surface and the length of the textile specimen are provided by the sample holder and the physical characteristics of the textile fabric.

## 3. Proposed System

In order to evaluate the DC volumetric electrical resistivity under different climatic conditions, we present in this work, on the one hand, a measurement system and on the other hand a methodology for obtaining this value under different climatic conditions. The measurement system stands on the technique previously described called “parallel bars”. The system consists of the following parts:

- *Static voltmeter*: this device will be in charge of applying the voltage to the fibre in order to DC volumetric electrical resistivity. This device will be connected to the computer to automate this process.
- *Climatic chamber equipped with sensors and actuators*: this device will establish the climatic conditions of the tests. This device is equipped with humidity and temperature sensors in order to continuously control these two characteristics inside of the chamber. Also, this climatic chamber should be equipped with actuators for decreasing and increasing both temperature and humidity; this will enable to control this parameter along the test is performed.
- *Control and data acquisition software*: this part of the system has two main modules, a sensing module (1), in charge of collect measurements of temperature and relative humidity from the chamber together with voltage values from the static voltmeter and time measurement of the test; and a control module (2) in charge of controlling the temperature and relative humidity of the climatic chamber and the needed actions over the static voltmeter in order to perform the tests. This software will export all the data for further data analysis.

**Figure 4:**
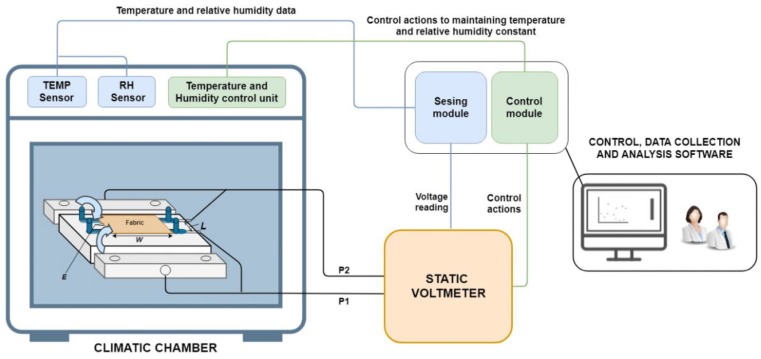
General diagram of the proposed measurement system.

The main components of the proposed measurement system and their interconnections are shown in Figure 4. 

Together with the previous measurement system, we present the methodology for obtaining the resistivity of each tissue under test.

The first step in the methodology is to choose the tissue under test and to analyse their fabric characteristics such as ligament, thickness, laminar dough and tissue density. This information could be useful for further analysis of the obtained results after the test.

Secondly, in order to obtain reliable and reproducible results, a cleaning process of the tissue must be done. This will enable to eliminate impurities present in the tissue before the study.

Then, the tissue must be adapted to the measures of the equipment’s holder. For obtaining reproducible results, five samples of the tissue will be cut for the analysis. The further steps in the methodology will be applied sequentially to these five samples.

Next, these samples will then be placed on each sample holder and introduced into the climatic chamber for an initial 24-h climate adaptation in the initial climatic conditions of the test.

**Figure 5:**
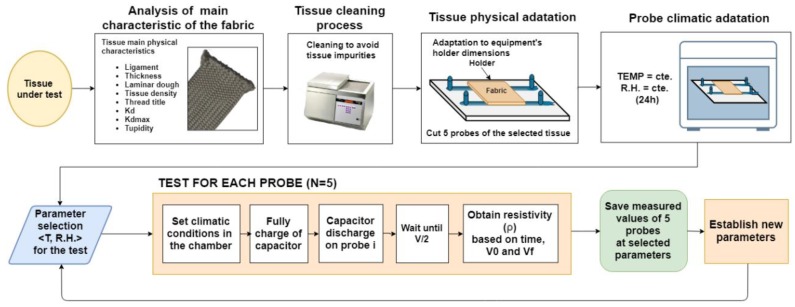
Block diagram of the methodology used for the tests.

Once the initial climatic adaptation is performed, the test will begin, and it will be necessary to establish which climatic variables will be constant and which ones will be modified (relative humidity and temperature). For each pair of climatic conditions (temperature–relative humidity) and each sample of the tissue, the previously described method of capacitor discharge through the static voltmeter is applied. This method consists of a first step where capacitor is fully charged, then the following step where this capacitor is discharged in the sample of tissue under test until the capacitor reaches its V/2 time value (the half-discharge time), through this value, the resistivity is obtained and the test for that sample finish at these temperature and relative humidity values. This process is performed for each sample in order to obtain a mean value of all these measures. All the data related to temperature, relative humidity, voltages and discharge time is monitored and controlled by the software during the test for further analysis. This process is performed for each pair of parameters (temperature – relative humidity), having a waiting time of two hours of adaptation to the new climatic conditions for each new pair of values. The main flowchart of the proposed methodology is shown in the Figure 5.

This methodology will allow us to analyse the behaviour of the resistivity of the tissue at different climatic conditions and obtain reliable results. In the next sections, a case study implementing the measuring system and the proposed methodology is described.

## 4. Case Study

To validate the proposed system and methodology, a case study is presented in which three tissue samples will be analyzed in the proposed measurement equipment and following the methodology presented above.

In order to develop the above measuring system, a device based on a static voltmeter has been built, and that is why the textile samples must initially be conditioned to the characteristics of this equipment and the environmental conditions of each test to be performed. 

**Figure 6:**
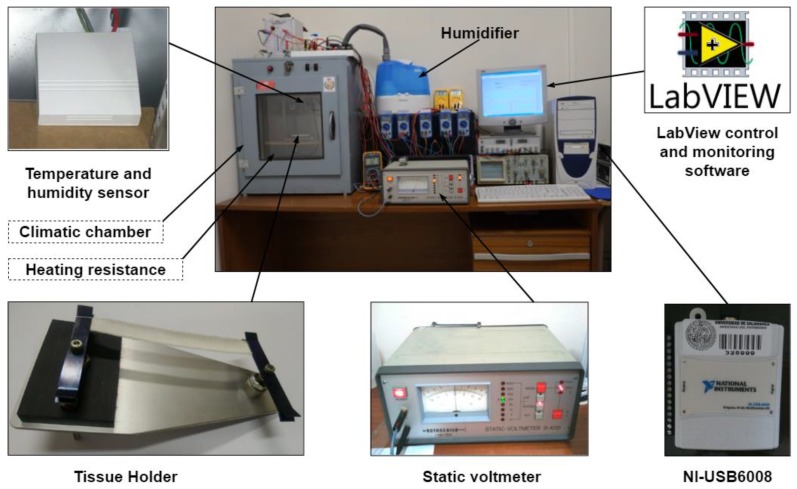
The fundamental components of the test equipment.

The characteristics of this equipment are those provided by each of the main elements and their interconnections. The static voltmeter is the R4021 model of the Rothschild [33] brand whose specific parameters for the tests are as follows: V0 = 145 V, Vf = 72.5 V, C = 8 pF and an initial capacitor charge time of 2.3 s. The climatic chamber has a volume of 64 dm^3^ and has been complemented with an HU 2060 ultrasonic humidifier of the Orbegozo brand and an LCN-FTW04 sensor [34], the first employed to humidify with small particles of water, approximately 3 µm in diameter, and the second for having more precise control of the environmental characteristics of the interior of the climatic chamber, with a maximum error of 3%. The computer equipment used has allowed controlling the rest of the equipment and the collection and processing of data through a designed LabVIEW [35] program. For the proper functioning of the equipment, it has been necessary to select the appropriate means for its interconnections, to avoid errors in the collection and measurement of the analysed parameters, being the National Instruments NI-USB6008 [36] data acquisition card the one used as a link between the climatic chamber, static voltmeter and the developed LabVIEW control and monitoring software. The fundamental components and their main interconnections for testing are shown in Figure 6.

Since the equipment is based on the static voltmeter model R4021, the textile samples must be adequately adapted to the holder of this device (left side of Figure 6) which is then placed inside of the climatic chamber. This promotes good reproducibility in the test results.

Since the measurements of the textile sample are irregular (10 cm long × 1 cm wide), we must recognise the two fundamental directions of the fabric, weft and warp, so that the cut of the five specimens gives identified and equal samples.

In this case study, the fundamental directions of the fabric have been considered in order to evaluate if these also affect the electrical behaviour under different climatic conditions. This analysis was carried out in three fabrics of different compositions (two of natural fibres and another of chemical fibres) in the textile laboratories of the Higher Technical School of Industrial Engineering of Béjar (University of Salamanca).

The names of the three fabrics chosen for the study are: cotton fabric (composed of 100% natural cotton fibers), woolen fabric (composed of 100% wool natural fibers) and technical fabric (composed of different chemical fibres and that is why we will call it technical fabric, 93% Nomex, 5% Kevlar and 2% antistatic fibre) (Table 1).

As indicated above, the main addresses have been identified, the properties in the warp direction being those indicated in Table 2.

To be able to verify if there are differences in the electrical conductivity DC, we have chosen fabrics with different warp and weft characteristics, the characteristics in the latter direction mentioned being those reflected in Table 3.

## 5. Results and Discussion

For the verification of the formulated methodology, the initial tests were carried out on the pure cotton textile material in a direction of the fabric (warp), at a constant temperature (T) of 292 K and varying the percentage of relative humidity in the air (RH) from 27% to 70%. Once the methodology has been verified, tests have been performed on three fabrics with different textile materials and in the two fundamental directions of the fabric. The results obtained have made it possible to verify that in any textile material and in any of its measuring directions, the increase in environmental values considerably increases the DC electrical conductivity. 

### 5.1. Verifying the Cleaning Process of the Methodology 

**Figure 7:**
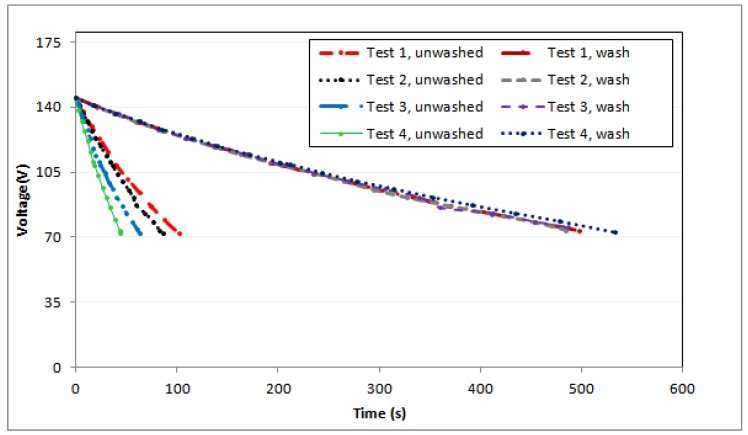
Discharge characteristics of the SATEN SUGAR fabric at a temperature of 292 K and a relative humidity of 31% with and without washing treatment.

The initial performed tests did not give good reproducibility in the results. Therefore, it was decided to perform a pre-tissue treatment consisting of a washing process in the Linitest with 2 mL/L of a non-ionic detergent, Sandozina MRN, for 30 min at a temperature of 45 °C with a bath ratio of 1/50 [37]. With this, a good reproducibility of the results has been obtained (Figure 7 shows the results of several tests with textile samples with and without prior treatment) and it has been concluded that these differences are due to the impurities of the tissues, which modify the electrical behavior, obtaining more disparate results at a lower relative humidity since the textile material has a lower intrinsic content in water.

Therefore, it was decided to include in the methodology an initial step of prior treatment consisting of a washing treatment to be able to eliminate any foreign matter or substance in the structure of the tissue that can modify its electrical behavior. 

### 5.2. Behavior of the DC Electrical Conductivity of the Three Textile Fabrics

To make a comparison of the behavior of the DC electrical conductivity in different tissues, the tests must be carried out under the same conditions, that is, the tissues must be washed, carried out in the same direction (this case, in warp), at a T constant of 292 K and a RH constant (different tests where the RH takes values between 27% to 70%). The absolute values of DC electrical resistivity to each environmental condition are given in Table A1, Table A2 and Table A3 of Appendix A, as well as the values of the fundamental parameters defined above. It is verified how the obtained values are decreasing, being able to adjust this lowering to a decreasing exponential curve, giving coefficients of determination [38], higher than 0.975. This gives the characteristic equation of each fabric, Table 4, in the warp direction and at a temperature of 292K. 

**Figure 8:**
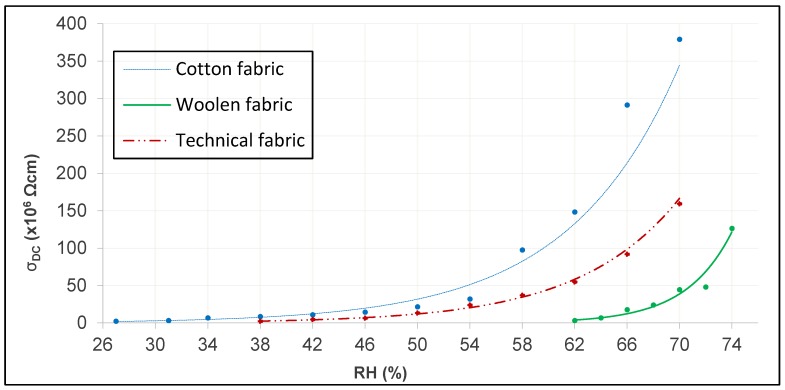
DC electrical conductivity according to RH.

Through these data, the decrease in DC electrical resistivity through its negative slopes becomes evident, the value of these slopes being different for each type of tissue. Therefore, there is an evidence that the increase in the relative humidity of the air will influence the DC electrical resistivity of the fabric, being more pronounced in the wool fabric than the other tissues studied. Since the parameter of DC electrical conductivity is the inverse of the DC electrical resistivity, the behavior will be the inverse and, therefore, increases with the increase of RH as can be seen in Figure 8. It is also observed that under the same environmental conditions, the fabric with the highest DC electrical conductivity is the cotton fabric, while the wool fabric has the lowest value.

**Figure 9:**
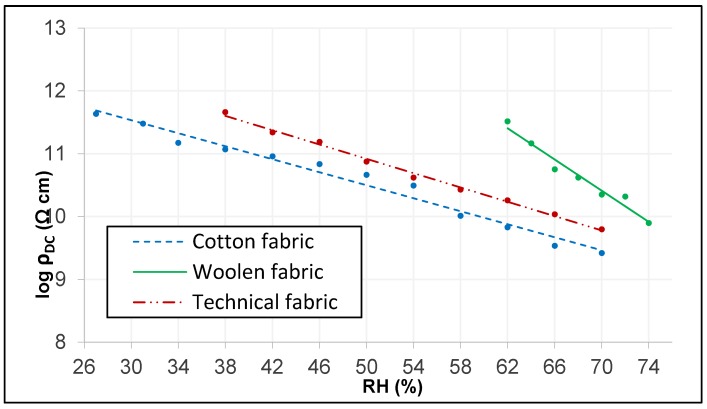
Linear adjustment of DC electrical resistivity.

It can be clearly seen in Figure 8 how the DC electrical conductivity of the different tissues have very small values at different RH, so the intervals chosen for each of them are different; from an RH of 27% for the cotton fabric, RH of 38% for the technical fabric and RH of 62% for the woolen fabric. In addition, it is observed how the change in the climatic conditions in the tissues affects the electrical conductivity of the material, being greater when the relative humidity of the air increases due to the increase of the water content in the tissue. Hearle et al. [39,40] obtained the intrinsic relation between relative humidity and electrical resistance through Equation (3).

(3) $$\log\;\left[R\right]\;=\;-a\;RH\;+\;b\;\to \;\log\;\left[\rho_{DC}\right]\;=\;-a\;RH\;+\;b,$$

where *RH* is the relative humidity in (%), *R* is the DC electrical resistance of the material in (Ω), ρ*_DC_* is the DC electrical resistivity of the material in (Ω cm), and both a and b are constant and dimensionless factors. Since the DC electrical resistance and the DC electrical resistivity are proportional, a linear adjustment is obtained that more easily determines the behavior of the variable depending on the RH as can be seen in Figure 9. Their values are shown in Table A4 of Appendix B.

As can be seen in Figure 9 and demonstrate through the values obtained from Equation (3) (Table A4 of Appendix B), the slope of the straight of the cotton fabric is the lowest, followed by that of the technical tissue and being the highest is wool. These values are consistent with the density and the Tupidity of the tissues, the relationship between these terms being as follows: the higher the density of the tissue, the lower the slope of the electrical resistivity of the air, and this is due to the lower possibility of inclusion of air water molecules between the threads of the tissue.

### 5.3. Behavior of the DC Electrical Conductivity According to the Direction of Measurement in the Textile Fabric

In the previous section we have studied the behavior of DC electrical conductivity as a function of the fibers that textile fabrics have, but another important parameter when evaluating this behavior is the direction in which the parameter is measured in the tissue. The manufacture of the fabric is done in two directions; warp and weft. In this section the influence of the value of the DC electrical conductivity with respect to the measurement direction will be evaluated, performing the tests in the three tissues studied previously. 

The absolute values of DC the electrical resistivity, as well as the values of the fundamental parameters defined above for each environmental condition are given in Table A1, Table A2 and Table A3 of Appendix A for the warp direction and in Table A6, Table A7 and Table A8 of Appendix C for the frame direction. It is verified how the obtained values are decreasing, being able to adjust this decrease to a decreasing exponential curve, giving coefficients of determination, R^2^, higher than 0.975. With this, the characteristic equations in the weft direction (Table 5) are obtained at a temperature of 292 K of each fabric.

**Figure 10:**
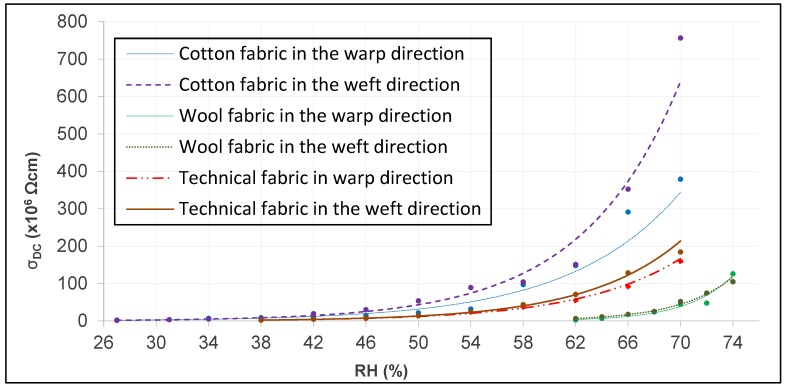
DC electrical conductivity according to RH in warp and weft.

Through the comparison of all these data, the decrease of the DC electrical resistivity in both directions through its negative slopes becomes evident, the value of these slopes being similar in each tissue. However, each fabric provides different slope values under the same test conditions, that is, the influence of RH is different depending on the type of textile material. With this it can be concluded that although it seems that the density or denseness of the tissue in each direction can influence this value, these characteristics do not have a significant influence on the value of the DC electrical resistivity and, therefore, on the DC conductivity electric. Figure 10 shows the variation of the DC electrical conductivity for each tissue in each direction.

Through the intrinsic relationship provided by Equation (3), the linear adjustments of the three tissues in both directions are obtained. To perform the graphic analysis in the two main directions of the tissue, the analysis of the error bar by standard deviation is used, which indicates the variability of the plotted data.

**Figure 11:**
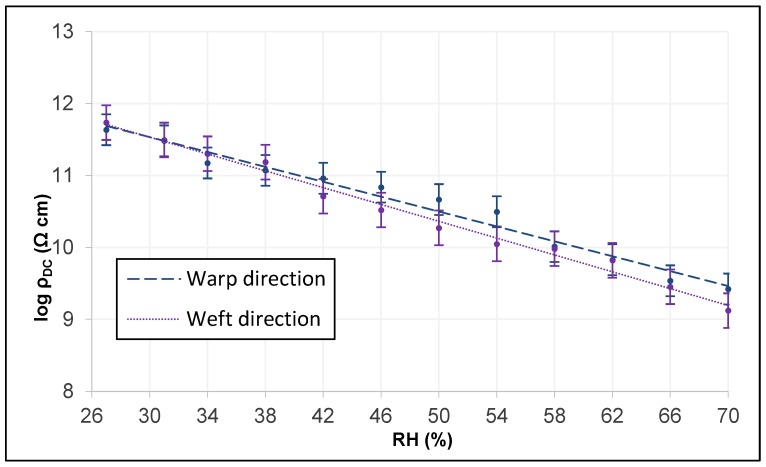
Linear adjustment of DC electrical resistivity in warp and weft of the cotton fabric.

Figure 11 shows those of the cotton fabric and reveals how they have very similar slopes, although the electrical conductivity of DC is somewhat more different from a higher RH, possibly because it has a greater thread title (tex) in the weft direction.

**Figure 12:**
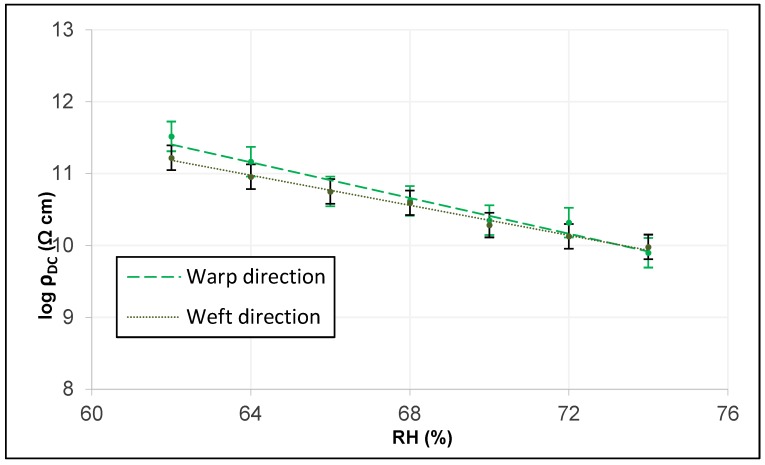
Linear adjustment of DC electrical resistivity in warp and weft of the woolen fabric.

Figure 12 shows those of the woolen fabric and reveals how they have very similar slopes, although the electrical conductivity of DC is somewhat more different from the lower RH, possibly because it does not have a higher thread title (tex) in the direction of the frame in the warp.

**Figure 13:**
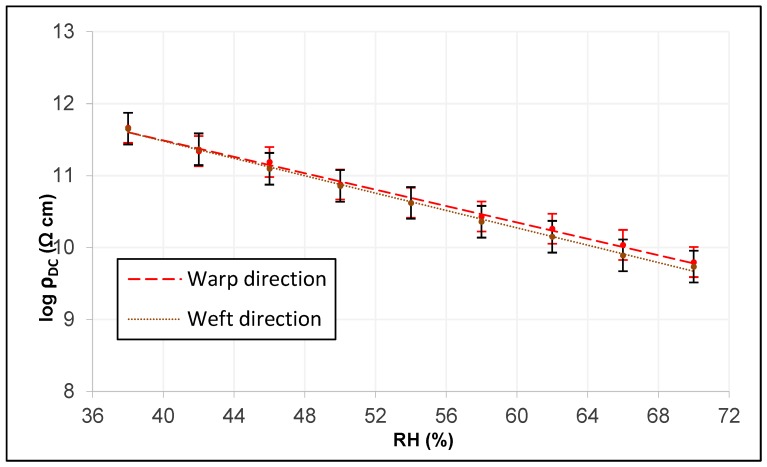
Linear adjustment of DC electrical resistivity in warp and weft of the technical fabric.

Figure 13 shows those of the technical fabric and reveals how they have very similar slopes, although the electrical conductivity of DC is somewhat more different from a higher RH, possibly because it has a greater thread title (tex) in the weft direction.

As can be seen through the values obtained from equation 3 (Table A4 and Table A5 of Appendix B), the slope of the lines in any direction of the cotton fabric is the lowest, followed by that of the technical fabric and the highest is wool. These values are correlated with the density and denseness of the tissues in both directions, being lower to greater density of the tissue in the direction studied.

No conclusive data can be drawn on the influence of the value of the continuous electrical resistivity with respect to the direction of the study (very similar slopes) and therefore more studies have to be carried out on a larger number of tissues.

## 6. Conclusions

This work has proposed and evaluated a methodology for obtaining the DC electrical conductivity of textile fabrics, and for this purpose, test equipment was designed based on this procedure. This equipment is composed of several elements whose purposes are based on several objectives: measurement, control of the test, capture of data and obtaining of results. For the test, the main equipment used are a static voltmeter, a climatic chamber and computer equipment with a developed measuring and control software. The equipment must be maintained in good maintenance conditions (such as the cleaning of the climatic chamber) to obtain a low rate of erroneous tests and thus perform the least discard of erroneous tests that have arisen (due to other circumstances, such as for example the self-discharge of the test piece), to minimize the time of tests since the preparation of these is important. However, it is versatile set-up and changing or incorporating new modules can allow the equipment evaluate different parameters of the materials - such as the tissue holder being able to obtain surface resistivity - and thus be able to expand results for future research.

Through the results achieved in the range of values set for each environmental characteristic, it can be concluded that the increase in the relative humidity of the air significantly increases the value of the DC “specific” (volumetric) electrical conductivity in all textile fabrics subject to test; with results varying exponentially from 2 to 756 pS/cm for the cotton fabric, from 5 to 227 pS/cm for the woolen fabric, and from 3 to 254 pS/cm for the technical fabric.

In addition, the DC electrical conductivity values obtained as a function of the measurement direction (warp/weft) have not given very different values, although it tends to be different from extreme RH depending on the ratio of the thread title in both directions, being lower at high RH and higher thread title; the values for a RH of 62% are as follows: the value in the warp cotton fabric is 148 pS/cm, while in the fabric it is 151 pS/cm, in the wool it is 5 pS/cm, in the warp and weft it is 11 pS/cm, in the warp knit fabric it is 75 pS/cm, and in the frame of 97 pS/cm.

Therefore, the results show that the climatic conditions have a significant impact on the DC electrical properties of the different fabrics, this being important when incorporating conductive wires in the fabric of a Smart textile itself since changes in the climatic conditions can cause changes in the DC electrical conductivity. This can cause problems in the transmission of energy between Smart textile devices, either due to the interruption of energy (breaks in the twisted circuits in the fabric) or due to the rise or fall of DC voltage (incorrect transmission of energy).

The study of these electrical properties through the proposed methodology is a preliminary step to the design of Smart textiles since it helps to discard materials for the manufacture of these textiles and even to choose the best material for their development.

Through the technique described in this article, several future lines of work are proposed, such as the possibility of evaluating other climatic conditions such as the electrical behavior of textile fabrics against different initial tensions, against changes in temperature at fixed RH.

## Figures and Tables

**Table 1:** Main characteristics of the fabrics.

	Cotton Fabric	Woolen Fabric	Technical Fabric
Ligament	Satin	Taffeta	Serge
Thickness	0.70 (mm)	0.39 (mm)	0.51 (mm)
Laminar dough	376 (g/m^2^)	126.2 (g/m^2^)	261.8 (g/m^2^)
Tissue density	46 (h/cm)	22 (h/cm)	28 (h/cm)

**Table 2:** Main characteristics of the fabrics in the warp direction.

	Cotton Fabric	Woolen Fabric	Technical Fabric
Thread title (Tex)	36	34	40
Kd	8.73	4.12	5.60
Kdtex	276.00	130.15	177.09
Kdmax	9.13	5.88	97.49
Tupidity (%)	95.57	70.07	74.80

**Table 3:** Main characteristics of the fabrics in the weft direction.

	Cotton Fabric	Woolen Fabric	Technical Fabric
Thread title (Tex)	64	34	55
Kd	6.58	3.50	5.63
Kdtex	208	110.79	177.99
Kdmax	7.59	5.88	97.49
Tupidity (%)	86.72	59.52	75.18

**Table 4:** Characteristic equation and coefficient of determination of DC electrical resistivity for each textile fabric studied in the warp direction.

Textile Fabric	Characteristic Equation	*R* ^2^
Cotton fabric	$\rho_{DC}$ = 1.2 × 10^13^ × e^−0.119RH^	0.976
Woolen fabric	$\rho_{DC}$ = 70 × 10^17^ × e^−0.286RH^	0.964
Technical fabric	$\rho_{DC}$ = 4 × 10^13^ × e^−0.131RH^	0.995

**Table 5:** Characteristic equation and coefficient of determination of DC electrical resistivity for each textile fabric studied in the weft direction.

Textile Fabric	Characteristic Equation	*R* ^2^
Cotton fabric	$\rho_{DC}$ = 2 × 10^13^ × e^−0.135RH^	0.988
Woolen fabric	$\rho_{DC}$ = 4 × 10^17^ × e−^0.246RH^	0.994
Technical fabric	$\rho_{DC}$ = 6 × 10^13^ × e^−0.139RH^	0.998

## Appendix A

**Table A1:** Values of DC electrical resistivity as a function of RH in the cotton fabric in the warp direction and a temperature of 292 K.

RH (%)	$t_{med}\;(s)$	*R* (Ω)	$\rho_{DC}\;(\Omega \mathrm{cm})$	$\sigma_{DC}\;(S/\mathrm{cm})$
27	711	616 × 10^11^	432 × 10^9^	2 × 10^−12^
31	499	433 × 10^11^	303 × 10^9^	3 × 10^−12^
34	245	213 × 10^11^	149 × 10^9^	7 × 10^−12^
38	194	168 × 10^11^	118 × 10^9^	8 × 10^−12^
42	151	131 × 10^11^	91 × 10^9^	11 × 10^−12^
46	113	98 × 10^11^	69 × 10^9^	15 × 10^−12^
50	76	66 × 10^11^	46 × 10^9^	22 × 10^−12^
54	52	45 × 10^11^	31 × 10^9^	32 × 10^−12^
58	17	15 × 10^11^	10 × 10^9^	97 × 10^−12^
62	11	10 × 10^11^	7 × 10^9^	148 × 10^−12^
66	6	5 × 10^11^	4 × 10^9^	292 × 10^−12^
70	4	4 × 10^11^	3 × 10^9^	379 × 10^−12^

**Table A2:** Values of DC electrical resistivity as a function of RH in the woolen fabric in the warp direction and a temperature of 292 K.

RH (%)	$t_{med}\;(s)$	*R* (Ω)	$\rho_{DC}\;(\Omega \mathrm{cm})$	$\sigma_{DC}\;(S/\mathrm{cm})$
62	542	470 × 10^11^	183 × 10^9^	5 × 10^−12^
64	241	209 × 10^11^	81 × 10^9^	12 × 10^−12^
66	93	80 × 10^11^	31 × 10^9^	32 × 10^−12^
68	78	67 × 10^11^	26 × 10^9^	38 × 10^−12^
70	37	32 × 10^11^	13 × 10^9^	80 × 10^−12^
72	34	29 × 10^11^	11 × 10^9^	86 × 10^−12^
74	13	11 × 10^11^	4 × 10^9^	227 × 10^−12^

**Table A3:** Values of DC electrical resistivity as a function of RH in the technical fabric in the warp direction and a temperature of 292 K.

RH (%)	$t_{med}\;(s)$	*R* (Ω)	$\rho_{DC}\;(\Omega \mathrm{cm})$	$\sigma_{DC}\;(S/\mathrm{cm})$
38	761	659 × 10^11^	336 × 10^9^	3 × 10^−12^
42	361	312 × 10^11^	159 × 10^9^	6 × 10^−12^
46	254	220 × 10^11^	112 × 10^9^	9 × 10^−12^
50	124	107 × 10^11^	55 × 10^9^	18 × 10^−12^
54	69	60 × 10^11^	31 × 10^9^	33 × 10^−12^
58	44	38 × 10^11^	20 × 10^9^	51 × 10^−12^
62	30	26 × 10^11^	13 × 10^9^	75 × 10^−12^
66	18	16 × 10^11^	8 × 10^9^	126 × 10^−12^
70	10	9 × 10^11^	5 × 10^9^	219 × 10^−12^

## Appendix B

**Table A4:** Coefficients of the linear adjustment of the DC electrical resistivity in the textile material in the warp direction and a temperature of 292 K.

Textile Fabric	a	b	
Cotton fabric	−0.1193	30.139	0.976
Woolen fabric	−0.2859	43.416	0.964
Technical fabric	−0.1311	31.381	0.995

**Table A5:** Coefficients of the linear adjustment of the DC electrical resistivity in the textile material in the weft direction and a temperature of 292 K.

Textile Fabric	a	b	
Cotton fabric	−0.1348	30.599	0.988
Woolen fabric	−0.2468	40.517	0.994
Technical fabric	−0.1391	31.682	0.997

## Appendix C

**Table A6:** Values of DC electrical resistivity as a function of RH in the cotton fabric in the weft direction and a temperature of 292 K.

RH (%)	$t_{med}\;(s)$	*R* (Ω)	$\rho_{DC}\;(\Omega \mathrm{cm})$	$\sigma_{DC}\;(S/\mathrm{cm})$
27	895	776 × 10^11^	543 × 10^9^	2 × 10^−12^
31	513	444 × 10^11^	311 × 10^9^	3 × 10^−12^
34	331	286 × 10^11^	201 × 10^9^	5 × 10^−12^
38	253	219 × 10^11^	153 × 10^9^	7 × 10^−12^
42	85	73 × 10^11^	51 × 10^9^	20 × 10^−12^
46	55	47 × 10^11^	33 × 10^9^	30 × 10^−12^
50	31	27 × 10^11^	19 × 10^9^	54 × 10^−12^
54	18	16 × 10^11^	11 × 10^9^	89 × 10^−12^
58	16	14 × 10^11^	10 × 10^9^	104 × 10^−12^
62	11	9 × 10^11^	7 × 10^9^	151 × 10^−12^
66	5	4 × 10^11^	3 × 10^9^	352 × 10^−12^
70	2	2 × 10^11^	1 × 10^9^	756 × 10^−12^

**Table A7:** Values of DC electrical resistivity as a function of RH in the woolen fabric in the weft direction and a temperature of 292 K.

RH (%)	$t_{med}\;(s)$	*R* (Ω)	$\rho_{DC}\;(\Omega \mathrm{cm})$	$\sigma_{DC}\;(S/\mathrm{cm})$
62	273	237 × 10^11^	92 × 10^9^	11 ×10^−12^
64	149	129 × 10^11^	50 × 10^9^	20 × 10^−12^
66	93	80 × 10^11^	31 × 10^9^	32 × 10^−12^
68	64	56 × 10^11^	22 × 10^9^	46 × 10^−12^
70	32	27 × 10^11^	11 × 10^9^	93 × 10^−12^
72	22	19 × 10^11^	7 × 10^9^	134 × 10^−12^
74	16	14 × 10^11^	5 × 10^9^	188 × 10^−12^

**Table A8:** Values of DC electrical resistivity as a function of RH in the technical fabric in the weft direction and a temperature of 292 K.

RH (%)	$t_{med}\;(s)$	*R* (Ω)	$\rho_{DC}\;(\Omega \mathrm{cm})$	$\sigma_{DC}\;(S/\mathrm{cm})$
38	738	639 × 10^11^	326 × 10^9^	3 × 10^−12^
42	382	331 × 10^11^	169 × 10^9^	6 × 10^−12^
46	205	177 × 10^11^	91 × 10^9^	11 × 10^−12^
50	119	103 × 10^11^	52 × 10^9^	19 × 10^−12^
54	69	60 × 10^11^	31 × 10^9^	33 × 10^−12^
58	38	33 × 10^11^	17 × 10^9^	60 × 10^−12^
62	23	21 × 10^11^	10 × 10^9^	97 × 10^−12^
66	13	11 × 10^11^	6 × 10^9^	177 × 10^−12^
70	9	8 × 10^11^	4 × 10^9^	254 × 10^−12^
